# Supplementary material for: Time-of-day defines NAD+ efficacy to treat diet-induced metabolic disease by synchronizing the hepatic clock in mice
Source: Nat Commun. 2023 Mar 27;14:1685. doi: 10.1038/s41467-023-37286-2 (PMC10043291; doi:10.1038/s41467-023-37286-2)
Supplement: Supplementary file 1 — Supplementary Information [file 41467_2023_37286_MOESM1_ESM.pdf]

Supplementary Information (Supplementary Figures 1-9)

Time-of-day defines NAD$^+$ efficacy to treat diet-induced metabolic disease by synchronizing the hepatic clock in mice

Quetzalcoatl Escalante-Covarrubias [1], Lucía Mendoza-Viveros[1,2]; Mirna González-Suárez[1], Román Sitten-Olea[1]; Laura A. Velázquez-Villegas[3], Fernando Becerril-Pérez[1], Ignacio Pacheco-Bernal[1], Erick Carreño-Vázquez[2], Paola Mass-Sánchez[1], Marcia Bustamante-Zepeda[1], Ricardo Orozco-Solís[2], Lorena Aguilar-Arnal[1]*

[1] Departamento de Biología Celular y Fisiología, Instituto de Investigaciones Biomédicas, Universidad Nacional Autónoma de México, 04510 Mexico City, Mexico.
[2]Laboratorio de Cronobiología y Metabolismo, Instituto Nacional de Medicina Genómica, 14610 Mexico City, Mexico.
[3]Departamento de Fisiología de la Nutrición, Instituto Nacional de Ciencias Médicas y Nutrición Salvador Zubirán, 14080 Mexico City, Mexico.

*Corresponding author: loreaguilararnal@iibiomedicas.unam.mx

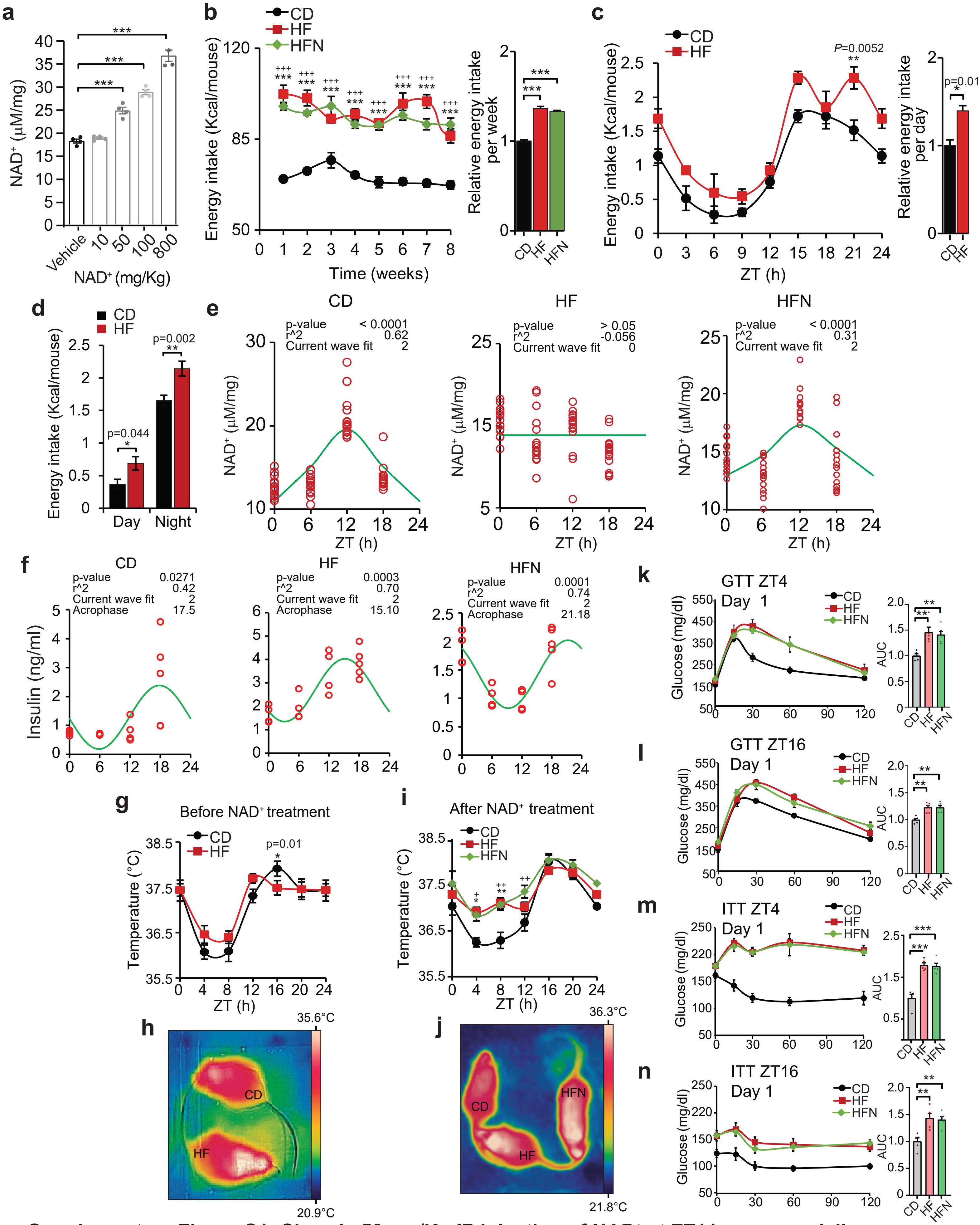

Supplementary Figure S1. Chronic 50 mg/Kg IP injection of NAD⁺ at ZT11 recovers daily rhythms in hepatic NAD⁺ in obese mice

a NAD⁺ was IP injected in mice at the indicated doses. One hour later, mice were sacrificed, and livers were snap frozen in liquid nitrogen. Hepatic NAD⁺ was quantified by HLPC (n = 3 mice and 3 technical replicates per dose). **b** Energy intake and relative energy intake per week (n = 20 mice per group) *(Continued on next page)*

c Energy intake throughout the day and relative energy intake per day (n = 4 mice per group).**d** Total energy intake during day and night periods (n =10 mice per group). **e** Daily rhythms in hepatic NAD^+ evaluated by CircWave. (n = 5 mice per time point and 3 technical replicates) **f** Rhythmic insulin levels evaluated with CircWave. **g** Circadian rectal temperature before NAD^+ treatment (n = 10 mice per group and 3 technical replicates) **h** Representative thermography image at ZT12 at week 8, before NAD^+ treatment (n = 4 mice per group). **i** Circadian rectal temperature 20 days after starting with NAD^+ treatment at ZT11 (n = 10 mice per group and 3 technical replicates) **j** Representative thermography image at ZT12, after 20 days on NAD^+ treatment at ZT11 (n = 4).**k-n** Glucose (k,l) and insulin (m,n) tolerance tests performed at both the rest (k, m; ZT4) and the active (l, n; ZT16) period the day before NAD^+ treatments (n = 5 mice, except for HFN in k, where n=6).
AUC: area under the curve. CD: Control diet fed mice; HF: High-fat diet fed mice; HFN: High-fat diet fed, NAD^+ treated mice at ZT11. Data represent mean ± SE, analyzed by two-way ANOVA using Tukey posttest, except for bar graphs, where one-way ANOVA followed by Tukey's posttest was used.
* $p < 0.05$, ** $p < 0.01$, *** $p < 0.001$. Statistical details and exact p values are provided in Supplementary Data 1. Symbol key for comparisons: * CD vs HF; + CD vs HFN; # HF vs HFN. Data from live mice were replicated in two independent experiments.

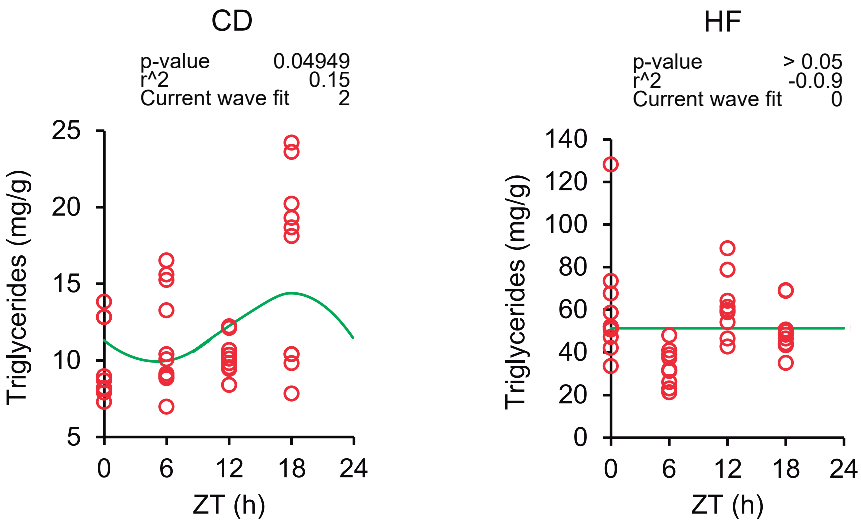

Supplementary Figure S2. Circadian rhythms in hepatic triglycerides

CircWave results for the investigations of circadian rhythmicity in hepatic triglycerides. CD: Control diet fed mice; HF: High-fat diet fed mice; HFN: High-fat diet fed, NAD^+ treated mice at ZT11. Circadian rhythmicity was confirmed when the CircWave *F* test produced a significant value (P<0.05). This test is one-sided.

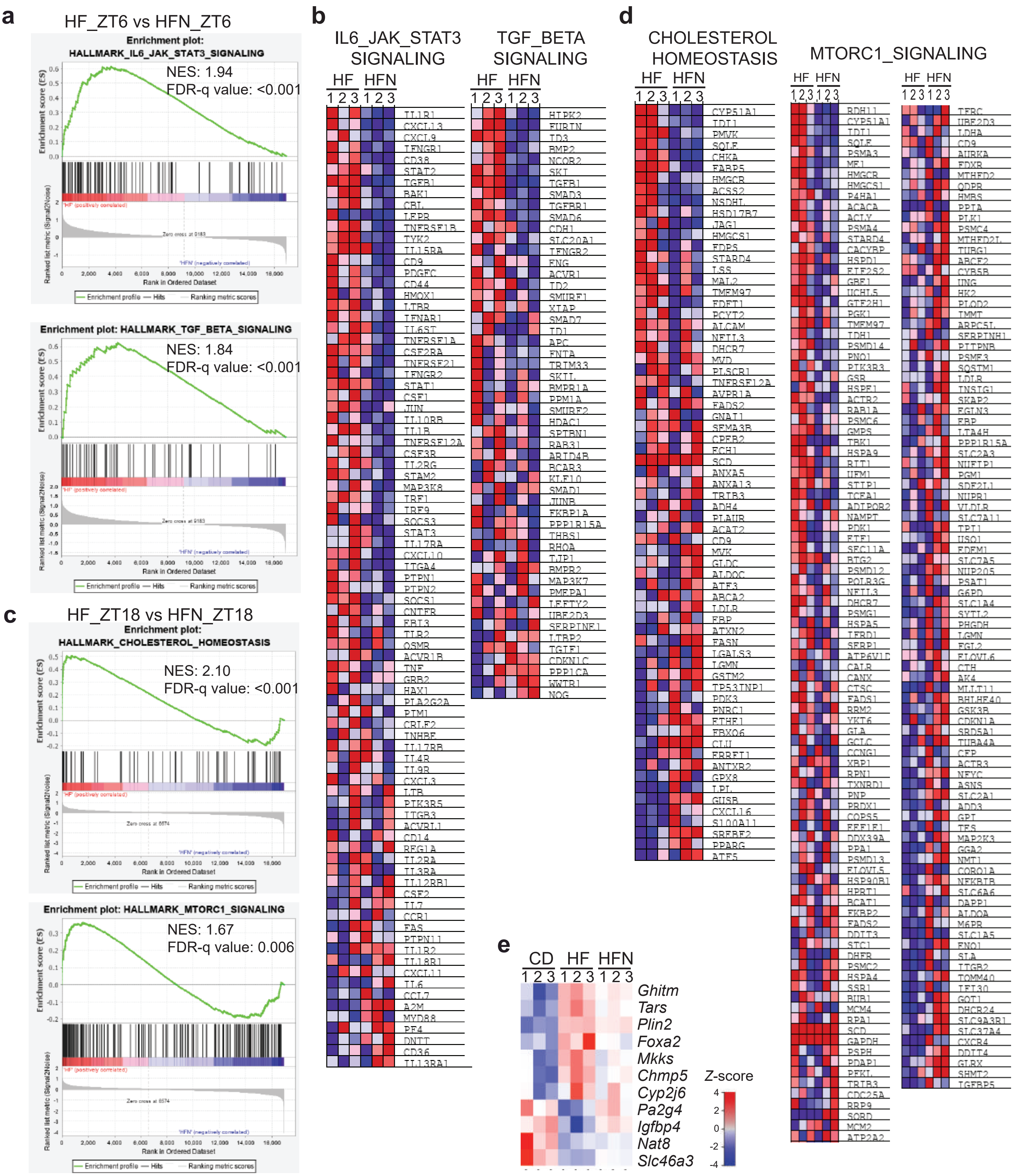

Supplementary Figure S3. Immune response and energy sensing pathways are corrected by a NAD⁺ chronotherapy in obese mice.

a, c Gene set enrichment analysis (GSEA) investigated within the molecular signature database (MSigDB) "Hallmark" gene set collection. Genes were rank-ordered by differential expression (DE) between obese mice untreated (HF) or treated with timed NAD⁺ therapy at ZT11 (HFN), specifically at the rest phase (A; ZT6)

or at the active phase (C; ZT18) **b, d** Heatmap depicting genes pertaining to the indicated gene set from the MSigDB, and rank-ordered according to their DE between HF and HFN groups, at ZT6 (b) or at ZT18 (d). *(Continued on next page)*

e Heatmap illustrating changes in expression from known target genes of FOXA2 (HNF3-β) transcription factor. NAD^+ chronotherapy elicits significant changes in expression from these genes. NES, normalized enrichment score; FDR, false discovery rate-adjusted q value.

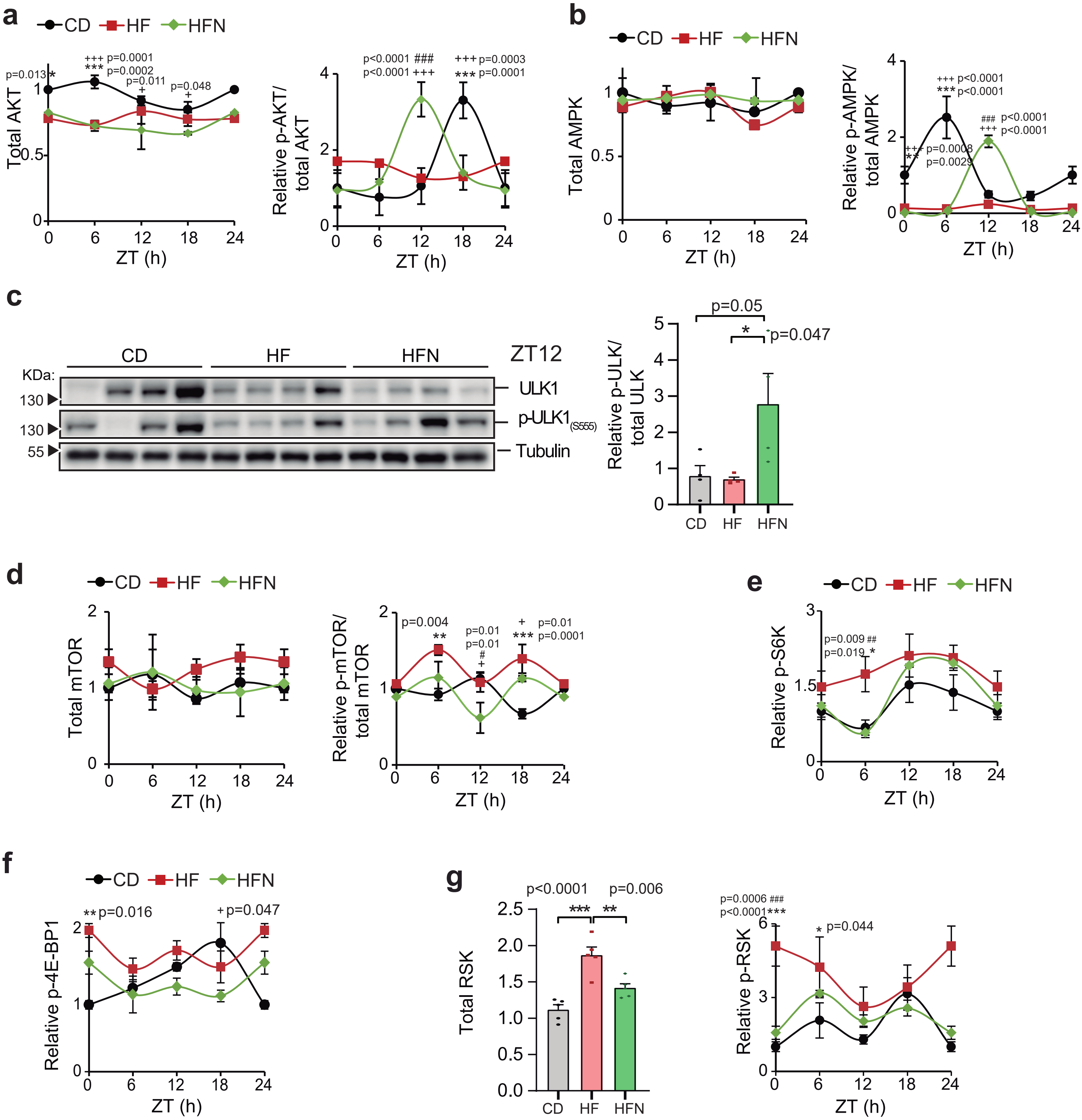

Supplementary Figure S4. Metabolic sensors regulated by NAD⁺ chronotherapy

a, b Quantification of western blots from n = 4 mice for the indicated proteins and relative phosphoprotein levels. All measurements were normalized to the tubulin loading control, and data from CD at ZT0 was set to 1 **c** Western blot for ULK1 and p-ULK1(S555) proteins from liver whole cell extracts sampled at ZT12 (left), and corresponding quantification by densitometry (right). Tubulin was used as loading control. n=4 **d-g** Quantification of western blots for the indicated proteins and relative phosphoprotein levels.d: n=4, e: n=3, g: n=5 for RSK and n=4 for pRSK. All measurements were normalized to their corresponding tubulin or p84 loading controls, and data from CD at ZT0 was set to 1.

CD: Control diet fed mice; HF: High-fat diet fed mice; HFN: High-fat diet fed, NAD⁺ treated mice at ZT11. Points at ZT24 are duplicates of ZT0 replotted to show 24-h trends. Data represent mean ± SE and were analyzed by two-way ANOVA using Tukey posttest, except for bar graphs, where one-way ANOVA followed by Tukey's posttest was used. * p <0.05, ** p <0.01, *** p <0.001.

Symbol key for comparisons: * CD vs HF; + CD vs HFN; # HF vs HFN.

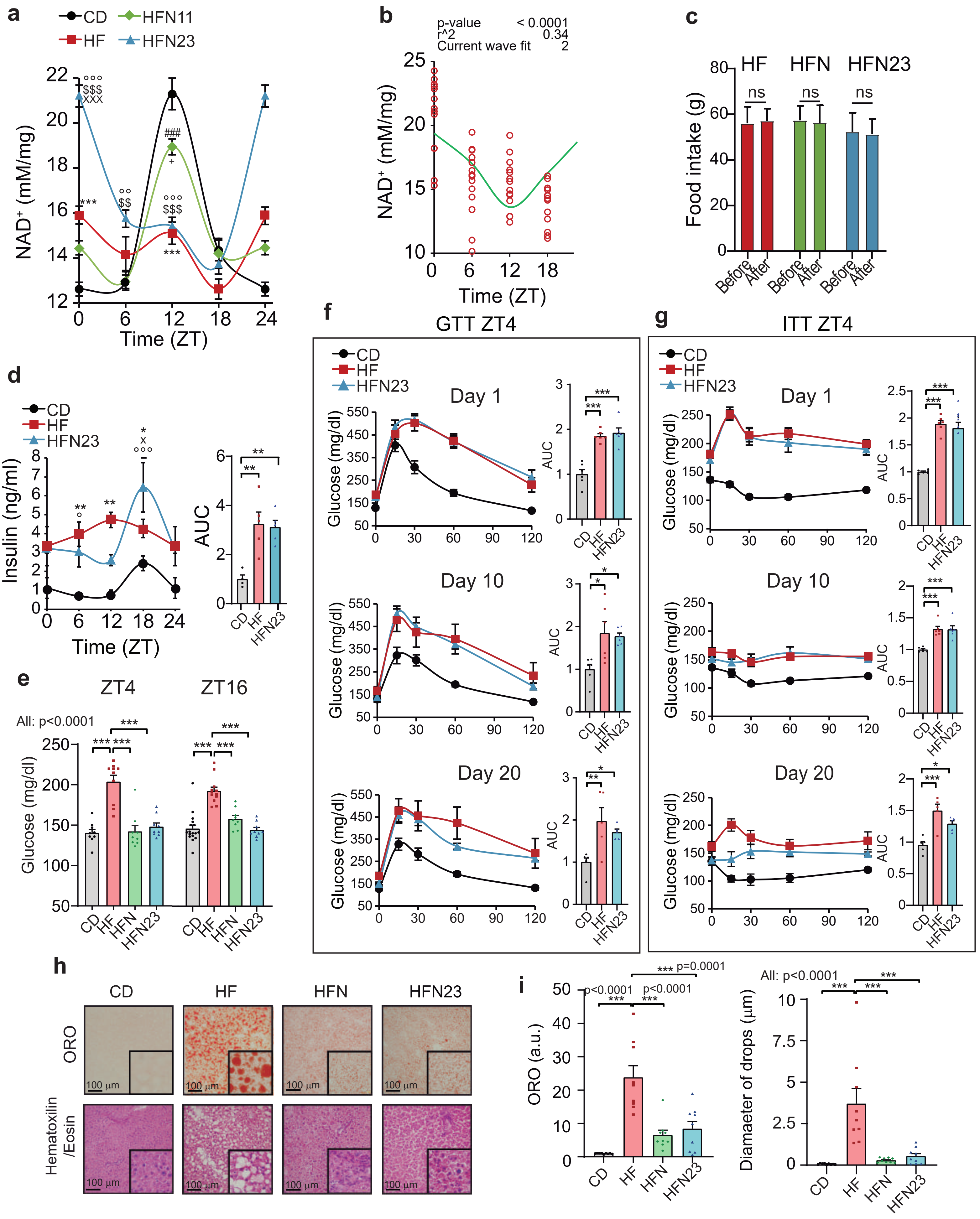

Supplementary Figure S5. Neutral lipid accumulation in the liver is relieved by NAD⁺ in obese mice independently of time of treatment.

a Hepatic NAD⁺ content measured by HPLC along the day at the indicated times for all groups after the experimental paradigm (n = 5 biological replicates, 3 technical replicates) **b** Circadian rhythms in hepatic NAD⁺ from mice treated with NAD⁺ at ZT23 (HFN23) was evaluated by CircWave. (n = 5 mice per time point and 3 technical replicates) *(Continued on next page)*

c Three weeks of food intake before and after the treatment were averaged for n= HF: 48; HFN: 37 ; HFN23: 27. Two-way ANOVA with Bonferroni post-test was applied, and the data are means ± SD **d** Circadian serum levels of insulin (n = 5 mice per time point and 2 technical replicates). AUC: area under the curve. **e** Circulating glucose levels were measured in fasted mice at ZT4 and at ZT16 (n=9, except for CD at ZT16 where n=17 and HF where n=12). **f, g** Glucose (f) and insulin (g) tolerance tests were performed at ZT4, on the day before NAD^+ treatments (day0), after 10 days of treatment (day 10) and at the end of the treatment (day20) (n = 6 for day 0 and day 10, and 5 for day 20). AUC: area under the curve.**h** Representative hepatic histopathology. Upper panel: Oil-red-O stain (ORO). Lower panel: Hematoxilin/Eosin. Images were acquired at 20X optical magnification, and detailed 100X magnification is shown. Data was reproduced in 3 biological and 3 technical replicates **i** Quantification of ORO signal in arbitrary units. Signal for control mice was set to 1. Length of lipid droplets was also compared between groups (n = 3 biological and 3 technical replicates).

CD: Control diet fed mice; HF: High-fat diet fed mice; HFN: High-fat diet fed, NAD^+ treated mice at ZT11; HFN23: High-fat diet fed, NAD^+ treated mice at ZT23. Points at ZT24 are duplicates of ZT0 replotted to show 24-h trends Data represent mean ± SE and were analyzed by one-way ANOVA followed by Tukey’s posttest. * $p < 0.05$, ** $p < 0.01$, *** $p < 0.001$. Statistical details and exact p values are provided in Supplementary Data 1.

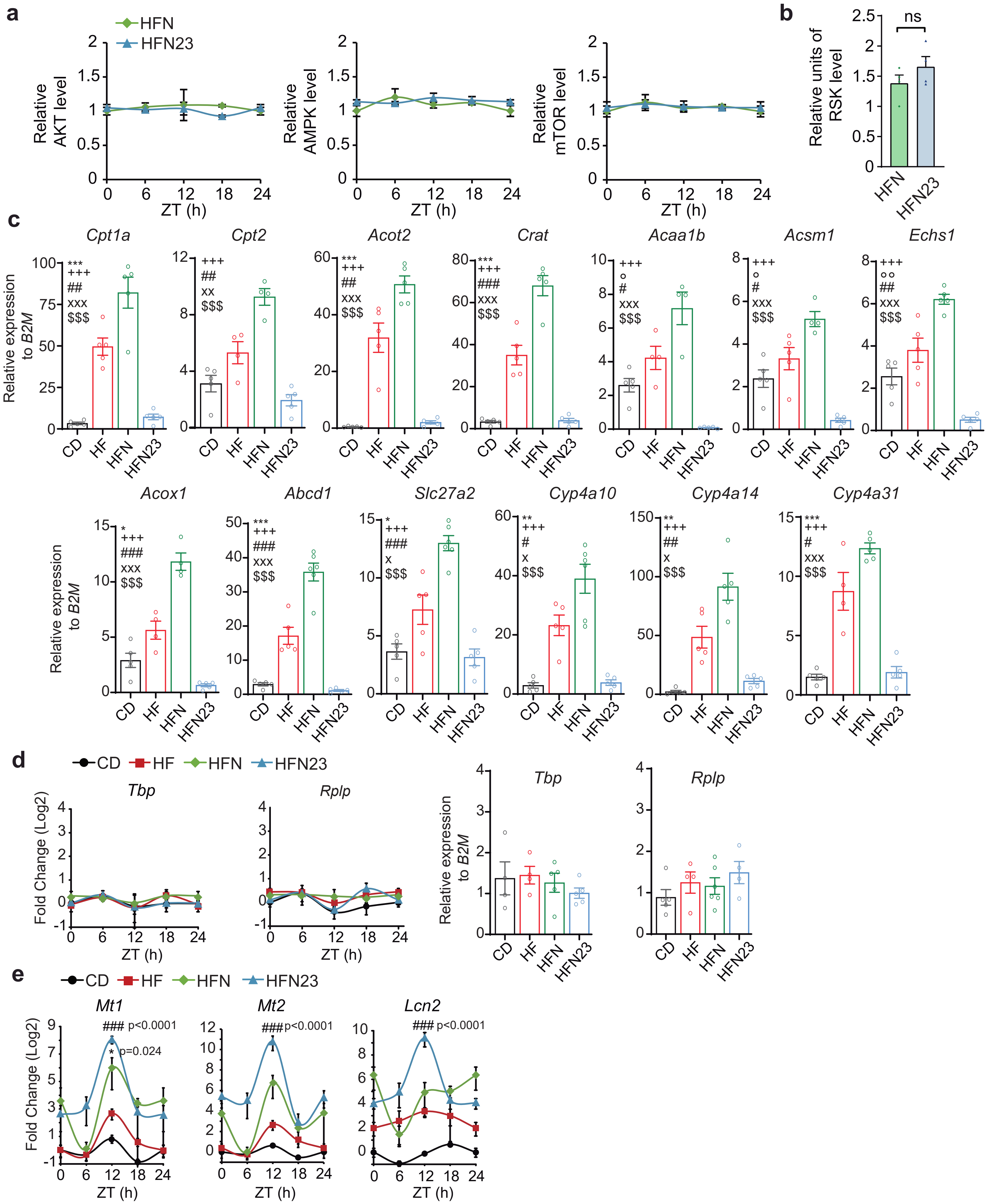

Supplementary Figure S6. Daily levels of nutrient sensors and gene expression profiles in the liver.

a Quantification of western blots from liver (n = 4 mice for HFN and 5 for HFN23) for the indicated proteins across the day. All measurements were normalized to their corresponding GAPDH or p84 loading control, and data from CD at ZT0 was set to 1. **b** Quantification by densitometry from western blots for RSK protein in liver whole cell extracts (n = 4). Two-tailed Student´s *t* test; n.s: non-significant.

(Continued on next page)

c RT-qPCR expression data in the liver for the indicated genes at ZT18 (n= 5 biological replicates per data point, except for HFN, where n=6). **d** RT-qPCR was used to assess hepatic expression of housekeeping genes across the day (CD, n=5; HF, n=4 except for ZT0, where n=5; HFN, n=6 except for ZT6, where n=5; HFN23, n=5 except for ZT12, where n=4). **e** RT-qPCR was used to determine expression of anti-obesity genes in the liver form mice at the indicated times-of-day (ZT) (n as in d). The data are means ± SE. *$p < 0.05$, **$p < 0.01$, ***$p < 0.001$, Two-way ANOVA followed by Tukey's post test. Statistical details and exact p values are provided in Supplementary Data 1. Points at ZT24 are duplicates of ZT0 replotted to show 24-h trends.
Symbol key for multiple comparisons: *CD vs HF, + CD vs HFN, °CD vs HFN23, # HF vs HFN, x HF vs HFN23, $ HFN vs HFN23.

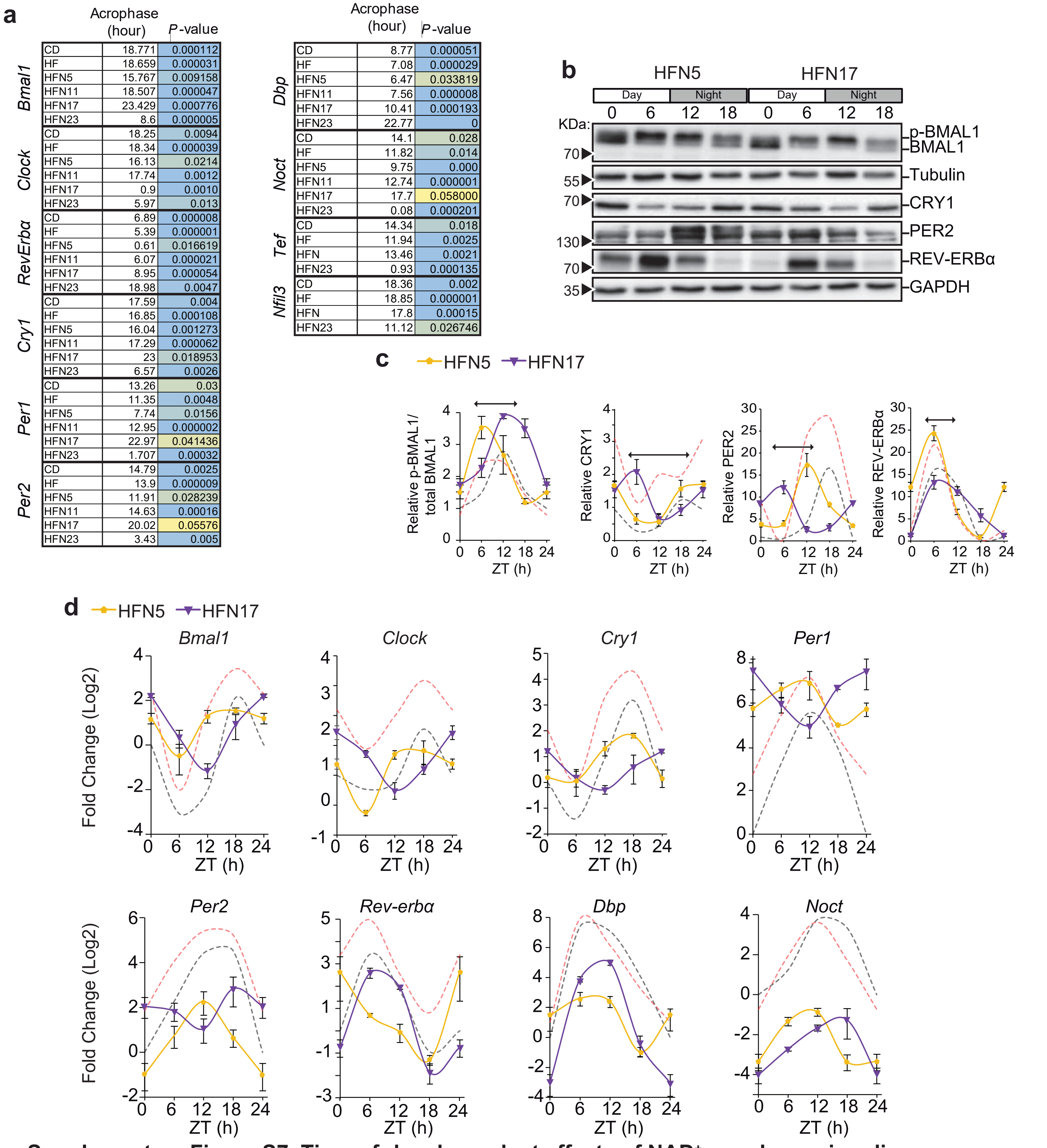

		Acrophase (hour)	*P*-value
Bmal1	CD	18.771	0.000112
	HF	18.659	0.000031
	HFN5	15.767	0.009158
	HFN11	18.507	0.000047
	HFN17	23.429	0.000776
	HFN23	8.6	0.000005
Clock	CD	18.25	0.0094
	HF	18.34	0.000039
	HFN5	16.13	0.0214
	HFN11	17.74	0.0012
	HFN17	0.9	0.0010
	HFN23	5.97	0.013
RevErbα	CD	6.89	0.000008
	HF	5.39	0.000001
	HFN5	0.61	0.016619
	HFN11	6.07	0.000021
	HFN17	8.95	0.000054
	HFN23	18.98	0.0047
Cry1	CD	17.59	0.004
	HF	16.85	0.000108
	HFN5	16.04	0.001273
	HFN11	17.29	0.000062
	HFN17	23	0.018953
	HFN23	6.57	0.0026
Per1	CD	13.26	0.03
	HF	11.35	0.0048
	HFN5	7.74	0.0156
	HFN11	12.95	0.000002
	HFN17	22.97	0.041436
	HFN23	1.707	0.00032
Per2	CD	14.79	0.0025
	HF	13.9	0.000009
	HFN5	11.91	0.028239
	HFN11	14.63	0.00016
	HFN17	20.02	0.05576
	HFN23	3.43	0.005

		Acrophase (hour)	*P*-value
Dbp	CD	8.77	0.000051
	HF	7.08	0.000029
	HFN5	6.47	0.033819
	HFN11	7.56	0.000008
	HFN17	10.41	0.000193
	HFN23	22.77	0
Noct	CD	14.1	0.028
	HF	11.82	0.014
	HFN5	9.75	0.000
	HFN11	12.74	0.000001
	HFN17	17.7	0.058000
	HFN23	0.08	0.000201
Tef	CD	14.34	0.018
	HF	11.94	0.0025
	HFN	13.46	0.0021
	HFN23	0.93	0.000135
Nfil3	CD	18.36	0.002
	HF	18.85	0.000001
	HFN	17.8	0.00015
	HFN23	11.12	0.026746

Supplementary Figure S7. Time-of-day dependent effects of NAD⁺ supply on circadian gene expression.

a Acrophase and p-value for circadian rhythmicity from clock and clock-controlled gene expression was assessed by CircaWave. **b** Circadian clock protein expression from liver whole cell extracts of obese mice treated with NAD⁺ at ZT5 (HFN5) or ZT17 (HFN17). Tubulin or GAPDH were used as loading control. **c** Quantification of western blots from n = 3 mice. Measurements were normalized to the loading control, and data from CD at ZT0 was set to 1. Averaged data from CD and HF mice are provided as reference, indicated as black (CD) or HF (red) dashed lines. Black arrows show the phase differences in the circadian wave between HFN5 and HFN17 mice. The data are means ± SE. **d** RT-qPCR determined circadian gene expression in the liver (n = 3 biological replicates per data point). The data are means ± SE. Averaged data from CD and HF mice are provided as reference, indicated as black (CD) or red (HF) dashed lines.

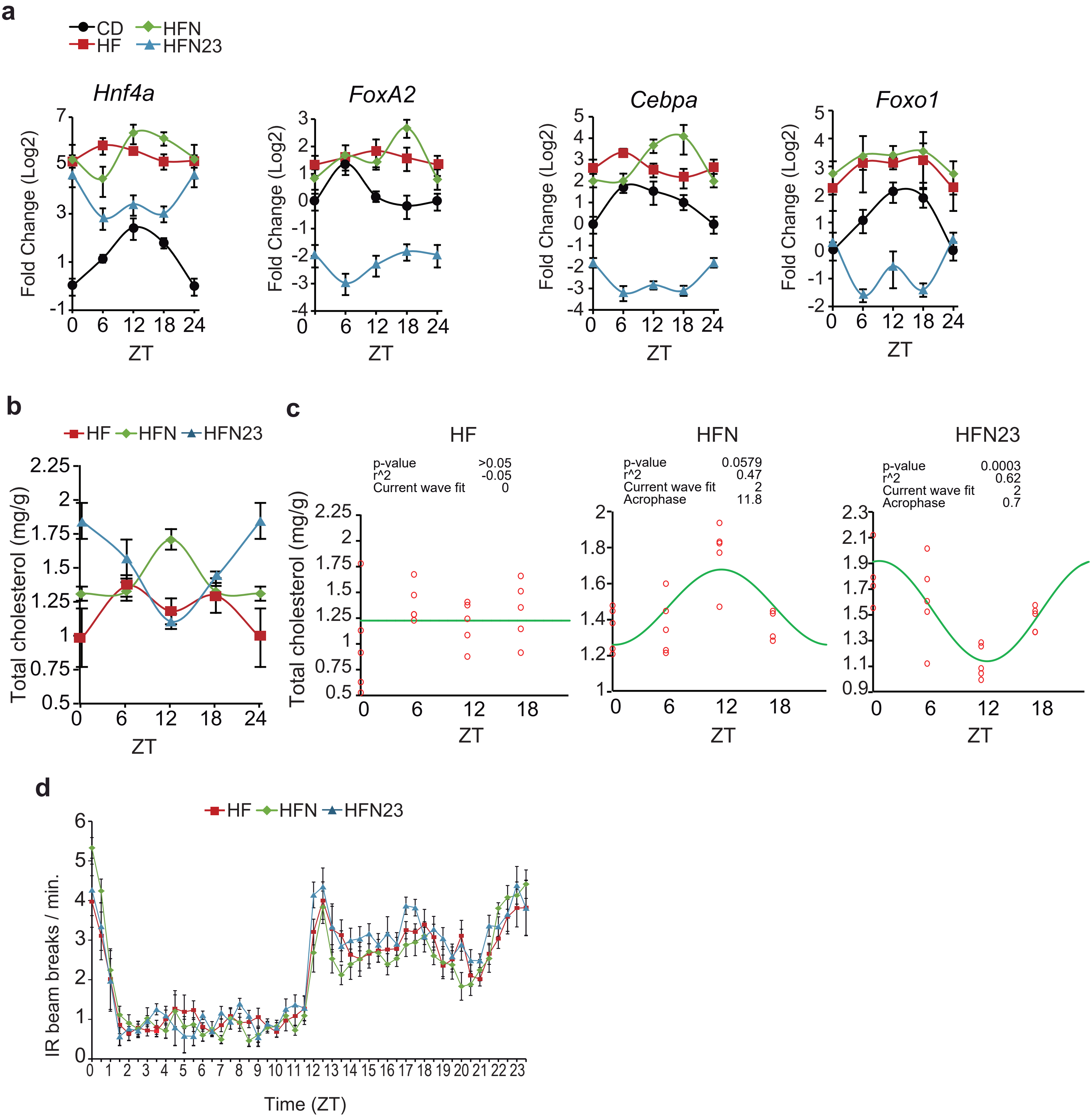

Supplementary Figure S8. Time-of-day dependent effects of NAD⁺ supply on rhythmicity of hepatic lipid metabolism.

a RT-qPCR was used to determine expression of genes related to lipid metabolism in the liver form mice at the indicated times-of-day (ZT) (per time point, n = 5 mice for CD and HF, 6 for HFN and 5 for HFN23 except at ZT6, where n=6). **b** Total cholesterol was measured in livers from the indicated groups of mice, at selected ZTs (n= 5 mice per data point, except for HFN at ZT18, where n=4). mg of cholesterol per gram of liver are plotted. **c** CircaWave analyses of rhythmicity from the hepatic cholesterol data. **d** Average 24-hour activity profile from the indicated groups of mice. Average was calculated for five days before NAD⁺ treatment. n= 7 mice for HF and HFN, and 5 for HFN23. CD: Control diet fed mice; HF: High-fat diet fed mice; HFN: High-fat diet fed, NAD+ treated mice at ZT11; HFN23: High-fat diet fed, NAD⁺ treated mice at ZT23. Points at ZT24 are duplicates of ZT0 replotted to show 24-h trends Data represent mean ± SE.

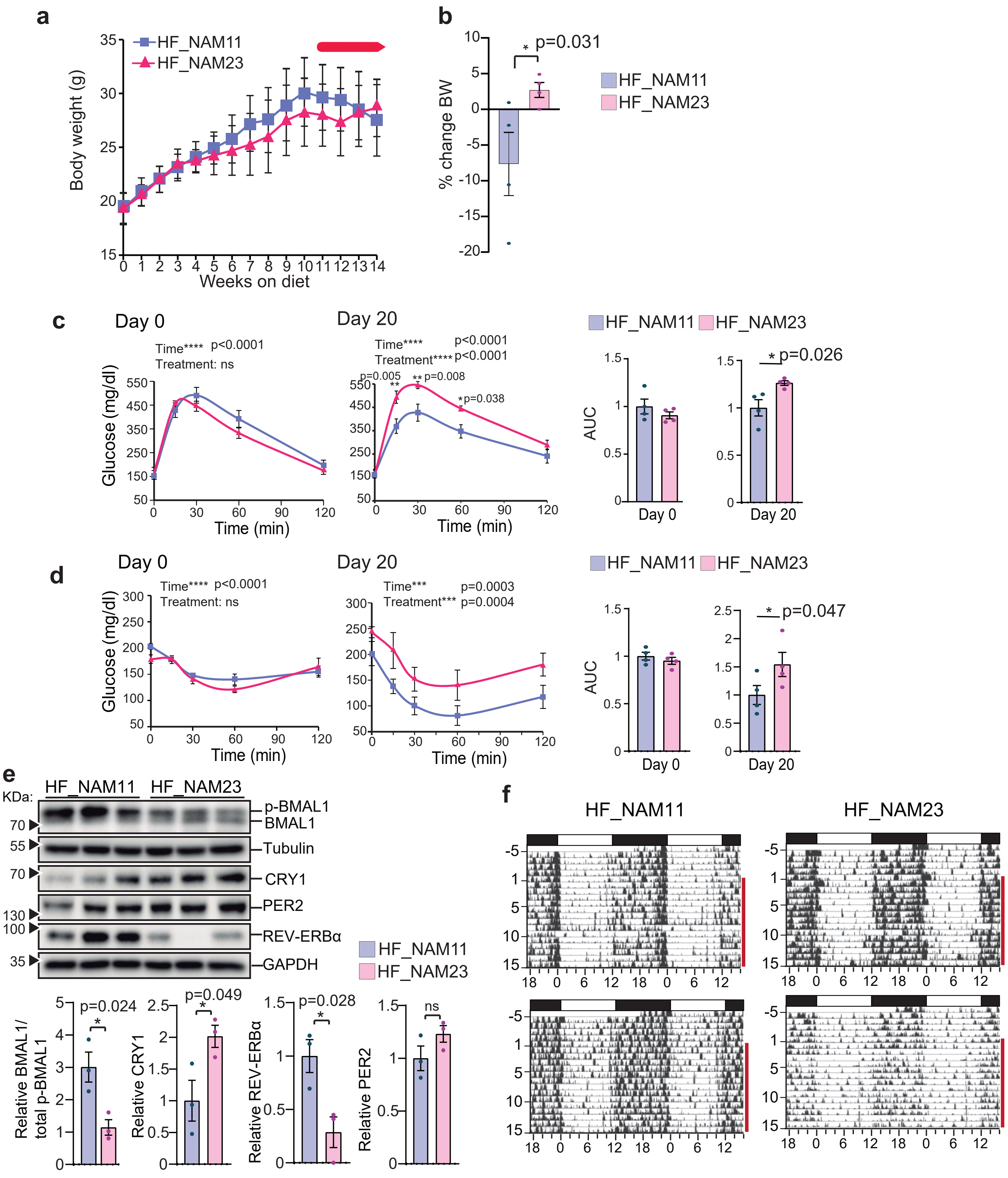

Supplementary Figure S9. Time-of-day dependent effects of the NAD⁺ precursor nicotinamide (NAM) to treat diet-induced obesity.

a Female mice fed a high-fat diet were IP injected at week 8, with 200 mg/Kg of NAM either at ZT11 (HF_NAM11) or at ZT23 (HF_NAM23) for three weeks. Weekly body weight measurements are shown (n = 4 mice per group). Red arrow indicates the period of treatment with NAM. **b** Percent change in body weight between weeks 8 (just before treatment), and 11 (end of the treatment). Two tailed *t*-test with n= 4 mice per group. **c, d** Glucose (GTT) and insulin (ITT) tolerance tests were performed at ZT4 before (day 0) and after (day 20) NAM treatments (n = 4 mice). AUC: Area under de curve. Two tailed *t*-test. *(Continued on next page)*

e Clock protein expression at ZT12 was measured by western blot from liver whole cell extracts of obese mice treated with NAM at ZT11 (HF_NAM11) or ZT23 (HF_NAM23). Tubulin or GAPDH were used as loading control. Quantification from n=3. Two tailed *t*-test **f** Representative double plotted actograms of locomotion measured using infrared sensors in a 12- hour light/12-hour dark cycle. The data represent means ± SE.
