# Supplementary material for: Time-of-day defines NAD+ efficacy to treat diet-induced metabolic disease by synchronizing the hepatic clock in mice
Source: Nat Commun. 2023 Mar 27;14:1685. doi: 10.1038/s41467-023-37286-2 (PMC10043291; doi:10.1038/s41467-023-37286-2)
Supplement: Supplementary file 3 — Description of Additional Supplementary Files [file 41467_2023_37286_MOESM3_ESM.pdf]

Description of Additional Supplementary Files

File Name: Supplementary Data 1
Description: Complementary statistic details and exact p values are provided for Fig. 1, Fig. 2, Fig. 3, Fig. 5, Fig. 6, Fig. 7 and supplementary Figures 1, 5 and 6.

File Name: Supplementary Data 2
Description: List of DE genes between ZT6 and Z18, and their biological processes.

File Name: Supplementary Data 3
Description: List of common genes in comparisons SD-HF vs HF-HFN and their functional analyses.

File Name: Supplementary Data 4
Description: List of common genes in comparisons SD-HFN vs HF-HFN and their functional analyses.

File Name: Supplementary Data 5
Description: List of primers used in this study.
