# Supplementary material for: Time-of-day defines NAD+ efficacy to treat diet-induced metabolic disease by synchronizing the hepatic clock in mice
Source: Nat Commun. 2023 Mar 27;14:1685. doi: 10.1038/s41467-023-37286-2 (PMC10043291; doi:10.1038/s41467-023-37286-2)
Supplement: Supplementary file 9 — Reporting Summary [file 41467_2023_37286_MOESM9_ESM.pdf]

nature portfolio

Corresponding author(s): LORENA AGUILAR ARNAL

Last updated by author(s): Feb 19, 2023

Reporting Summary

Nature Portfolio wishes to improve the reproducibility of the work that we publish. This form provides structure for consistency and transparency in reporting. For further information on Nature Portfolio policies, see our Editorial Policies and the Editorial Policy Checklist.

Statistics

For all statistical analyses, confirm that the following items are present in the figure legend, table legend, main text, or Methods section.

n/a	Confirmed	
☐	☒	The exact sample size (*n*) for each experimental group/condition, given as a discrete number and unit of measurement
☐	☒	A statement on whether measurements were taken from distinct samples or whether the same sample was measured repeatedly
☐	☒	The statistical test(s) used AND whether they are one- or two-sided<br>*Only common tests should be described solely by name; describe more complex techniques in the Methods section.*
☒	☐	A description of all covariates tested
☐	☒	A description of any assumptions or corrections, such as tests of normality and adjustment for multiple comparisons
☐	☒	A full description of the statistical parameters including central tendency (e.g. means) or other basic estimates (e.g. regression coefficient) AND variation (e.g. standard deviation) or associated estimates of uncertainty (e.g. confidence intervals)
☐	☒	For null hypothesis testing, the test statistic (e.g. *F*, *t*, *r*) with confidence intervals, effect sizes, degrees of freedom and *P* value noted<br>*Give P values as exact values whenever suitable.*
☒	☐	For Bayesian analysis, information on the choice of priors and Markov chain Monte Carlo settings
☒	☐	For hierarchical and complex designs, identification of the appropriate level for tests and full reporting of outcomes
☒	☐	Estimates of effect sizes (e.g. Cohen's *d*, Pearson's *r*), indicating how they were calculated

Our web collection on statistics for biologists contains articles on many of the points above.

Software and code

Policy information about availability of computer code

Data collection

The ORO The images were captured with the Olympus camera DP70 system using the DPController v 1.1.1.65 software, coupled to a Olympus BX51 microscope with the DPManager software v. 1.1.1.71.
For activity assesment in live mice, beam break data was continuously recorded and compiled with the OASPAD20 (OMNIALVA) software, v2019

Data analysis

Surface of lipid droplets was quantified using the ImageJ software (v 1.53).
Protein bands were quantified by densitometric analysis using Image Studio Lite Version 5.0 software (LI-COR biosciences).
Thermnographic mages processing was performed using FLIR-Tools software v 5.13.17214 (2015 FLIR® Systems).
GraphPad Prism version 8.4.2.679 for Windows (GraphPad Software Inc., San Diego, CA, USA) and Excel (Microsoft Office 360 v2301) were used for statistical analyses and plotting.
24-hours period rhythms were assessed employing CircWave version 1.4.
Figures were assembled using Adobe Illustrator CC 2015 (Adobe Inc., San José, CA, USA).
qPCR primers were designed using Primer3web v4.1.0
For Extracellular Flux Analyses, the instrument control, data analysis and file management was performed with the Agilent Seahorse Wave v2.6 software.
Analysis of time-series data for activity inlive mice was performed with RhythmicAlly, an open source program using R, update from 17th September 2019 available at: https://github.com/abhilashlakshman/RhythmicAlly
The arrays were scanned in the GeneChip Scanner 3000 7G (Affymetrix) and the GeneChip Command Console Software (v 4.0.3) was used to obtain the .CEL intensity files. Normalized gene expression data (.CHP files) were obtained with the Transcriptome Analysis Console (TAC v4.0.1.36) software using default parameters. Changes in gene expression (± 1. fold-change; FDR-corrected p-value ≤0.05) were subjected to functional analyses using the "Compute Overlaps" tool to explore overlap with the CP (Canonical Pathways) and the GO:BP (GO biological

process) gene sets at the MSigDB (molecular signature database) v7.0. The tool is available at: https://www.gsea-msigdb.org/gsea/msigdb/annotate.jsp, and estimates statistical significance by calculating the FDR q-value. This is the FDR analog of the hypergeometric P-value after correction for multiple hypothesis testing according to Benjamini and Hochberg. Gene set enrichment analysis (GSEA) was performed using GSEA v. 4.0.3. 54 to determine the enrichment score within the Hallmark gene set collection in MSigDB v7.0159, selecting the Signal2Noise as the metric for ranking genes. The findMotifs.pl program in the HOMER software v 2.0 160 was used for motif discovery and enrichment,

For manuscripts utilizing custom algorithms or software that are central to the research but not yet described in published literature, software must be made available to editors and reviewers. We strongly encourage code deposition in a community repository (e.g. GitHub). See the Nature Portfolio guidelines for submitting code & software for further information.

Data

Policy information about availability of data

All manuscripts must include a data availability statement. This statement should provide the following information, where applicable:

- Accession codes, unique identifiers, or web links for publicly available datasets
- A description of any restrictions on data availability
- For clinical datasets or third party data, please ensure that the statement adheres to our policy

All data generated or analyzed during this study are included in this article (and its supplementary information files). Source data are provided with this paper. All gene expression data that support the findings of this study have been deposited in the National Center for Biotechnology Information Gene Expression Omnibus (GEO) and are accessible through the GEO Series accession number: GSE163865.
The ChIP-Atlas database can be accessed at: https://chip-atlas.org/ . The Investigate Mouse Gene Sets tool to compute overlaps with gene sets in MSigDB can be accessed at: http://www.gsea-msigdb.org/gsea/msigdb/mouse/annotate.jsp

Human research participants

Policy information about studies involving human research participants and Sex and Gender in Research.

Reporting on sex and gender	N/A
Population characteristics	N/A
Recruitment	N/A
Ethics oversight	N/A

Note that full information on the approval of the study protocol must also be provided in the manuscript.

Field-specific reporting

Please select the one below that is the best fit for your research. If you are not sure, read the appropriate sections before making your selection.

☒ Life sciences ☐ Behavioural & social sciences ☐ Ecological, evolutionary & environmental sciences

For a reference copy of the document with all sections, see nature.com/documents/nr-reporting-summary-flat.pdf

Life sciences study design

All studies must disclose on these points even when the disclosure is negative.

Sample size	We did not carry out predetermination of sample size. For most of the experiments, at least five independent biological replicates where anaylized. In some experiments such as western blot, the number of n is 3-4 depending on available material and wether samples can be processed reliably in parallel. We concluded that the sample size is enough to cover the biological variance when the data was highly reproducible.
Data exclusions	N/A
Replication	In vivo experiments were performed at least two independent times to confirm reproducibility. The rest of experiments were performed at least three independent times. All findings from replicate experiments were consistent and confirmed.
Randomization	Allocation of animals were random
Blinding	Investigators were not blinded during in vivo experiments as in most cases, the bodyweight difference between treatments is obvious. However, no subjective process was included in the processing and analyses of the experimental data. qPCR, HPLC, ChIP and biochemical

determination experiments were performed with samples just numbered as 1, 2, 3 etc. and experimenters were not aware of the identity of the samples.

Reporting for specific materials, systems and methods

We require information from authors about some types of materials, experimental systems and methods used in many studies. Here, indicate whether each material, system or method listed is relevant to your study. If you are not sure if a list item applies to your research, read the appropriate section before selecting a response.

Materials & experimental systems

n/a	Involved in the study
☐	☒ Antibodies
☒	☐ Eukaryotic cell lines
☒	☐ Palaeontology and archaeology
☐	☒ Animals and other organisms
☒	☐ Clinical data
☒	☐ Dual use research of concern

Methods

n/a	Involved in the study
☒	☐ ChIP-seq
☒	☐ Flow cytometry
☒	☐ MRI-based neuroimaging

Antibodies

Antibodies used

Antibodies used in this study were: From Cell Signaling: PPARg (2443), AKT (9272), Phospho-AKTSer473 (9271), AMPKα (5831), Phospho-AMPKαThr172 (50081), mTOR (2983), Phospho-mTORSer2448 (5536), Phospho-p70 S6KThr389 (9234), Phospho-4E-BP1Thr37/46 (2855), RSK1/RSK2/RSK3 (9355), Phospho-p90RSKSer359 (8753), REV-ERBα (13418), ULK1 (8054), Phospho-ULK1Ser555 (5869), all diluted 1:1000; from Santa Cruz: C/EBPα (SC-365318, 1:500); from Abcam: BMAL1 (Ab3350, 1:1000); from Alpha Diagnostics International: PER2 (PER21-A 1:2000); from Bethyl Laboratories: CRY1 (A302-614A 1:1000); from Sigma: α-Tubulin (T5168, 1:80000); from Genetex: GAPDH-HRP (GTX627408-01, 1:120000) and P84 (GTX70220-01, 1:1000) The secondary antibodies were Anti-rabbit IgG (Cell Signaling, 7074, 1:150000 for BMAL1, 1:10000 for Pparg and 1:80000 for the rest) or Anti-mouse IgG (Sigma I8765, 1:80000), conjugated to horseradish peroxidase. For ChIP assays, a combination of two anti BMAL1 antibodies: 1.25 µL rabbit anti-BMAL1 (ab3350, Abcam) and 2.5 µL rabbit anti-BMAL1 (ab93806, Abcam) in 900 µL final volume. Immunoprecipitations with 4 µL of normal mouse IgG (Sigma-Aldrich, Cat. No. 18765) were performed simultaneously in 900 µL final volume.

Validation

Anti PPARγ (81B8) Rabbit mAb #2443 from Cell Signaling, is validated for Western Blotting, Immunoprecipitation, Immunofluorescence, ChIP and ChIP-seq. Species Reactivity: Human, Mouse. 289 citations in https://www.cellsignal.com/products/primary-antibodies/pparg-81b8-rabbit-mab/2443
Anti Akt Antibody #9272 from Cell Signaling is validated for Western Blotting, Immunoprecipitation Immunofluorescence, Flow Cytometry. Species Reactivity: Human, Mouse, Rat, Hamster, Monkey, Chicken, D. melanogaster, Bovine, Dog, Pig, Guinea Pig. 25,810 citations in https://www.cellsignal.com/products/primary-antibodies/akt-antibody/9272
Anti Phospho-Akt (Ser473) Antibody #9271 is validated for Western Blotting, Immunoprecipitation Immunofluorescence, Flow Cytometry. Species Reactivity: Human, Mouse, Rat, Hamster, Monkey, D. melanogaster, Bovine, Dog. 26,956 citations in https://www.cellsignal.com/products/primary-antibodies/phospho-akt-ser473-antibody/9271
Anti AMPKα (D5A2) Rabbit mAb #5831 is validated for Western Blotting, Immunoprecipitation. Species Reactivity: Human, Mouse, Rat, Monkey, Bovine. 607 citations in https://www.cellsignal.com/products/primary-antibodies/ampka-d5a2-rabbit-mab/5831
Anti Phospho-AMPKα (Thr172) (D4D6D) Rabbit mAb #50081 is validated for Western Blotting, Immunoprecipitation. Species Reactivity: Human, Mouse, Rat. 171 citations at https://www.cellsignal.com/products/primary-antibodies/phospho-ampka-thr172-d4d6d-rabbit-mab/50081
Anti mTOR (7C10) Rabbit mAb #2983 is validated for Western Blotting, Immunoprecipitation, Immunohistochemistry (Paraffin), Immunofluorescence (Immunocytochemistry), Flow Cytometry. Species Reactivity: Human, Mouse, Rat, Monkey. 2394 citations in https://www.cellsignal.com/products/primary-antibodies/mtor-7c10-rabbit-mab/2983?_=1676937327817&Ntt=mTOR&tahead=true
Anti Phospho-mTOR (Ser2448) (D9C2) XP® Rabbit mAb #5536 is validated for Western Blotting, Immunoprecipitation. Species Reactivity: Human, Mouse, Rat, Monkey. 1811 citations in https://www.cellsignal.com/products/primary-antibodies/phospho-mtor-ser2448-d9c2-xp-rabbit-mab/5536
Anti Phospho-p70 S6 Kinase (Thr389) (108D2) Rabbit mAb #9234 is validated for Western Blotting. Species Reactivity: Human, Mouse, Rat, Monkey. 1652 citations in https://www.cellsignal.com/products/primary-antibodies/phospho-p70-s6-kinase-thr389-108d2-rabbit-mab/9234
Phospho-4E-BP1 (Thr37/46) (236B4) Rabbit mAb #2855 is validated for Western Blotting, Immunohistochemistry (Paraffin), Immunofluorescence (Immunocytochemistry), Flow Cytometry. Species Reactivity: Human, Mouse, Rat, Monkey. 2280 citations in https://www.cellsignal.com/products/primary-antibodies/phospho-4e-bp1-thr37-46-236b4-rabbit-mab/2855?_=1676937777579&Ntt=2855&tahead=true
Anti RSK1/RSK2/RSK3 (32D7) Rabbit mAb #9355 is validated for Western Blotting, immunoprecipitation. Species Reactivity: Human, Mouse, Rat, Monkey, Guinea Pig. 116 citations in https://www.cellsignal.com/products/primary-antibodies/rsk1-rsk2-rsk3-32d7-rabbit-mab/9355
Anti Rev-Erbα (E1Y6D) Rabbit mAb #13418 is validated for Western Blotting, immunoprecipitation. Species Reactivity: Human, Mouse, Rat. 38 citations in https://www.cellsignal.com/products/primary-antibodies/rev-erba-e1y6d-rabbit-mab/13418?_=1676938090964&Ntt=13418&tahead=true
Anti ULK1 (D8H5) Rabbit mAb #8054 is validated for Western Blotting, immunoprecipitation. Species Reactivity: Human, Mouse, Rat. 625 citations in https://www.cellsignal.com/products/primary-antibodies/ulk1-d8h5-rabbit-mab/8054
Anti Phospho-ULK1 (Ser555) (D1H4) Rabbit mAb #5869 is validated for Western Blotting, immunoprecipitation. Species Reactivity: Human, Mouse. 340 citations in https://www.cellsignal.com/products/primary-antibodies/phospho-ulk1-ser555-d1h4-rabbit-mab/5869?_=1676938289268&Ntt=5869&tahead=true

C/EBP α mouse mAb (D-5): sc-365318 is validated for western blotting, immunoprecipitation, immunofluorescence, ELISA. Species Reactivity for human and mouse. 57 citations in https://www.scbt.com/es/p/c-ebp-alpha-antibody-d-5#citations
Anti-BMAL1 antibody (ab3350) is a Rabbit polyclonal to BMAL1, validated for: WB, ICC, Reacts with: Mouse, Rat, Human. 63 references in https://www.abcam.com/bmal1-antibody-ab3350.html
Anti-BMAL1 antibody (ab93806) is a Rabbit polyclonal to BMAL1, validated for: WB, IP, Reacts with: Mouse, Human. 56 references are available at: https://www.abcam.com/bmal1-antibody-ab93806.html
Rabbit Anti-Mouse Per 2 IgG # 1(aff pure) Cat # PER21-A from Alpha Diagnostics is validated for western blotting, ELISA, histochemistry. Species Reactivity for mouse and rat. 17 citations in https://www.labome.com/product/Alpha-Diagnostics/PER21-A.html
p84 antibody [5E10] (HRP) from Genetex is a mouse monoclonal (5E10) validated for western blot in Human, Mouse, Rat, Hamster, Monkey. References for this clone are available in: https://www.genetex.com/Product/Detail/Nuclear-Matrix-Protein-p84-antibody-5E10/GTX70220#references
GAPDH antibody [GT239] (HRP) from Genetex is a mouse monoclonal (GT239) validated for WB, IHC-P. Reactivity: Human, Mouse, Rat, Hamster . References are available in https://www.genetex.com/Product/Detail/GAPDH-antibody-GT239-HRP/GTX627408-01#references

Mouse monoclonal Anti-α-Tubulin antibody (T5168) clone B-5-1-2, from SIGMA is validated for western blot from mouse. 3388 publications are available in https://www.sigmaaldrich.com/MX/es/search/t5168?focus=papers&page=1&perpage=30&sort=relevance&term=t5168&type=citation_search
Cry1 Antibody, Rabbit Polyclonal Cat. No. A302-614A is validated for western blotting, immunoprecipitation in human, however, multiple publications validate it for western blot from mouse extracts, as shown in multiple citations found at: https://www.citeab.com/antibodies/657012-a302-614a-rabbit-anti-cry1-antibody-affinity-purifie

Animals and other research organisms

Policy information about studies involving animals; ARRIVE guidelines recommended for reporting animal research, and Sex and Gender in Research

Laboratory animals	Four-week-old C57Bl/6J mice were obtained from the Biological Models Unit at the Instituto de Investigaciones Biomédicas (UNAM, Mexico). The mice were kept under a 12:12-h light:dark cycles. Food and water were provided ad libitum. Temperature and humidity were constantly monitored.
Wild animals	N/A
Reporting on sex	Female mice were used for the experiments using NAM as a NAD+ precursor, and the rest of the experiments were performed in male mice.
Field-collected samples	N/A
Ethics oversight	All animal experimental procedures were reviewed and approved by the Internal Committee for the Care and Use of Laboratory Animals (CICUAL) at the Instituto de Investigaciones Biomédicas, (UNAM, Mexico), and are registered under protocol no. ID240.

Note that full information on the approval of the study protocol must also be provided in the manuscript.
